# Elimination of Cancer Cells in Co-Culture: Role of Different Nanocarriers in Regulation of CD47 and Calreticulin-Induced Phagocytosis

**DOI:** 10.1021/acsami.2c19311

**Published:** 2023-01-12

**Authors:** Eman M. Hassan, Samantha McWhirter, Gilbert C. Walker, Yadienka Martinez-Rubi, Shan Zou

**Affiliations:** † Metrology Research Centre, National Research Council Canada, 100 Sussex Drive, Ottawa, OntarioK1A0R6, Canada; ‡ Department of Chemistry, University of Toronto, 80 St. George St., Toronto, OntarioM5S3H6, Canada; § Security and Disruptive Technologies, National Research Council Canada, 100 Sussex Drive, Ottawa, OntarioK1A0R6, Canada; ∥ Department of Chemistry, Carleton University, 1125 Colonel By Drive, Ottawa, OntarioK1S5B6, Canada

**Keywords:** CD47, siRNA, cytotoxicity, cancer, downregulation, GO, BNNT, LNP

## Abstract

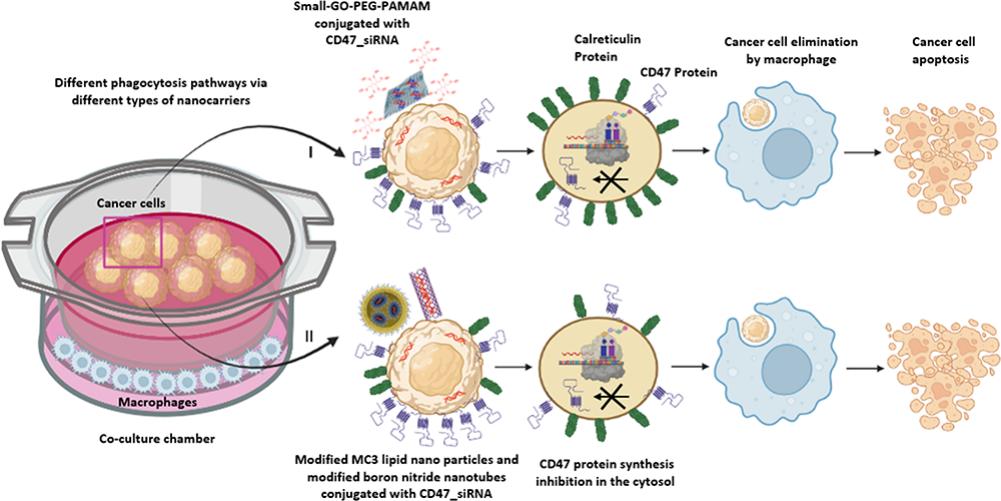

Under healthy conditions, pro- and anti-phagocytic signals are balanced. Cluster of Differentiation 47 (CD47) is believed to act as an anti-phagocytic marker that is highly expressed on multiple types of human cancer cells including acute myeloid leukemia (AML) and lung and liver carcinomas, allowing them to escape phagocytosis by macrophages. Downregulating CD47 on cancer cells discloses calreticulin (CRT) to macrophages and recovers their phagocytic activity. Herein, we postulate that using a modified graphene oxide (GO) carrier to deliver small interfering RNA (siRNA) CD47 (CD47_siRNA) in AML, A549 lung, and HepG2 liver cancer cells in co-culture *in vitro* will silence CD47 and flag cancer cells for CRT-mediated phagocytosis. Results showed a high knockdown efficiency of CD47 and a significant increase in CRT levels simultaneously by using GO formulation as carriers in all used cancer cell lines. The presence of CRT on cancer cells was significantly higher than levels before knockdown of CD47 and was required to achieve phagocytosis in co-culture with human macrophages. Lipid nanoparticles (LNPs) and modified boron nitride nanotubes (BNPs) were used to carry CD47_siRNA, and the knockdown efficiency values of CD47 were compared in three cancer cells in co-culture, with an achieved knockdown efficiency of >95% using LNPs as carriers. Interestingly, the high efficiency of CD47 knockdown was obtained by using the LNPs and BNP carriers; however, an increase in CRT levels on cancer cells was not required for phagocytosis to happen in co-culture with human macrophages, indicating other pathways’ involvement in the phagocytosis process. These findings highlight the roles of 2D (graphene oxide), 1D (boron nitride nanotube), and “0D” (lipid nanoparticle) carriers for the delivery of siRNA to eliminate cancer cells in co-culture, likely through different phagocytosis pathways in multiple types of human cancer cells. Moreover, these results provide an explanation of immune therapies that target CD47 and the potential use of these carriers in screening drugs for such therapies *in vitro*.

## Introduction

Tumor cells are able to escape immunosurveillance through several distinct mechanisms, one of which is the upregulation of the CD47 anti-phagocytic signal.^1^ CD47 is a transmembrane protein overexpressed by acute myeloid leukemia (AML) and several types of solid tumors.^2^ The binding of CD47 to its receptor signal regulatory protein α (SIRPα) on macrophages serves as a signal inhibiting the phagocytosis of tumor cells. A blockade of CD47 by therapeutic monoclonal antibodies disrupts CD47-SIRPα interaction and leads to phagocytosis in AML and solid tumors.^1,3,4^ Upon the blockade of CD47, tumor cells are shown to display a pro-phagocytic signal on their surfaces to be phagocytosed.^5^ Calreticulin (CRT) is a chaperone protein located in the endoplasmic reticulum (ER). It is involved in calcium homeostasis in the ER and other functions related to protein folding.^6,7^ CRT was shown to translocate to the cell membrane of normal and tumor cells during apoptosis, where it functions as an “eat me” signal, promoting phagocytosis.^8^ It has been reported that CRT is upregulated on both the surface of AML and on solid tumors after administrating the anti-CD47 antibody.^5^ We have previously shown the selective elimination of AML cells in co-culture with lipopolysaccharide (LPS)-activated macrophages via downregulation of CD47 and upregulation of CRT simultaneously.^9^ However, the connection between CD47 and CRT is also controversial. It was observed that phagocytosis was only decreased upon CRT blockade in macrophages (as opposed to being decreased after blocking CRT on cancer cells).^10^ This different findings could be related to different expression levels of CRT and CD47 that were required by using dissimilar types of cellular systems to study phagocytosis in different labs. Whether CRT and CD47 could individually contribute to the regulation of phagocytosis deserves further exploration, which may provide a different and better design for non-CD47-mediated CRT-based therapy. Using similar or the same types of cellular model systems with controlled and regulated expression levels of CRT and CD47 may provide a new route for understanding the connection and individuality of these markers.

Small interfering RNA (siRNA) is a short double-stranded RNA with 21–23 nucleotides. It suppresses protein synthesis via the rapid degradation of the target mRNA and subsequently reduces the corresponding protein level.^11^ In the past several years, siRNA technology has witnessed rapid development and became one of the most recent and revolutionary approaches used in gene therapy, especially in cancer research with multiple drug resistance.^12−14^ However, the delivery of siRNA to cells faces many challenges including sensitivity to enzymatic degradation, fast clearance, immunogenicity, and incapability of reaching to the targeted sites.^12^ To address these issues, an effective carrier is needed. Due to their numerous advantages, nanocarriers have gained significant interest for drug delivery in the past decade.^15^ They have the ability to protect siRNA from enzymatic degradation and improve cellular penetration, and they can be synthesized or assembled to have uniform sizes and shapes to improve cellular delivery.^16,17^ Many nanocarriers have been reported to carry siRNA in different types of cancers and effectively knock down the gene of interest.^17−19^ In this study, graphene oxide (GO), lipid nanoparticles (LNPs), and boron nitride nanotubes (BNNTs) as nanocarriers are compared for the delivery of CD47_siRNA in cancer cells and their knockdown efficiency values are monitored.

GO, a two-dimensional (2D) nanomaterial, has recently attracted extensive interest for biosensing applications.^20,21^ GO is obtained by exfoliating high-purity graphite into single-layered sheets or flakes. Thanks to the abundant hydrophilic surface groups (such as hydroxyl and carboxyl groups), GO can be dispersed in water.^22^ The presence of such groups makes it easy to functionalize the surface of GO flakes with biocompatible polymers, which provide high biocompatibility and loading capacity as carriers.^23,24^ Moreover, coating GO flakes with protein and polyethylene glycol (PEG) makes their *in vitro* or *in vivo* toxicity negligible.^25^ Therefore, functionalized GO has been developed into ideal gene delivery systems.^20^ GO and its derivatives have been used as carriers to deliver siRNA in many types of solid tumors, such as pancreatic,^26^ breast,^27^ and cervical cancers.^28^ We have reported the successful use of different GO formulations to deliver CD47_siRNA to AML cells *in vitro*.^29^ GO was synthesized and processed at several sizes and modified with PEG and dendrimers (PAMAM). Successful knockdown of CD47 was obtained with minimal toxicity in AML cells *in vitro.*

Lipid nanoparticles are the most commonly used non-viral vectors for siRNA delivery. Many types of lipids have been developed to improve transfer efficiency and stability and decrease the immune response and renal escape of siRNA.^30,31^ The most popular type is the ionizable amino lipid, which plays a major role in protecting siRNAs and facilitating their cytosolic transport. Moreover, ionizable lipids can be positively charged at acidic pH to condense siRNAs into LNPs but are neutral at physiological pH, which decreases toxicity.^32^ The first approved siRNA drug by Food and Drug Administration (FDA) was delivered in a lipid nanoparticle using an ionizable amino lipid, DLin-MC3-DMA (MC3), to treat hereditary transthyretin amyloidosis (hATTR).^33^

BNNTs are a structural analog of carbon nanotubes in which alternating B and N atoms are substituted for C atoms. BNNTs have many interesting properties, such as high resistance to oxidation, high radiation absorption, and thermal conductivity. Therefore, they have been used in a wide range of applications, including tissue engineering, sensing, and gene delivery applications.^34−36^ However, their high hydrophobicity and chemical stability make them difficult to disperse in aqueous media for medical applications. Therefore, physical and chemical functionalization approaches are used to disperse BNNTs in aqueous solutions. For instance, BNNTs are derivatized with poly-l-lysine,^37^ glycol-chitosan,^38^ polyimide,^39^ and poly(*p*-phenylene-ethynylene).^40^ We have also carried out extensive studies on the self-assembly of polythiophene on BNNTs in organic solvents^41−43^ and nanocomposite fabrics.^44^

To explore the subsequent effect of CD47 downregulation on CRT levels and tumor cell elimination by macrophages, the knockdown of CD47 was carried out by the delivery of CD47_siRNA to AML, lung, and liver tumor cells *in vitro* using different carriers with different modification strategies, namely, 2D flakes of GO modified with PEG and dendrimers (GO-PEG-PAMAM), 1D nanotubes functionalized with water-soluble polymers (BNP: BNNT-polymer), and “0D” nanoparticles using ionizable lipids (LNPs). The knockdown efficiency of CD47 protein, changes of CRT levels, and phagocytosis of tumor cells by macrophages were investigated and compared among the nanocarriers with different dimensions and possibly different delivery routes. With different carriers and delivery strategies, different levels of CRT and CD47 expression levels could be achieved using the same types of cancer cell systems. Studying the dynamic relationship between CD47 “don’t eat me” signal and CRT “eat me” signal could be used as a platform to screen potential drugs against AML and solid tumors, especially for those that gain drug resistance.

## Results and Discussion

### Transfection of CD47_siRNA in Cancer Cells Using Modified Graphene Oxide as a Nanocarrier in Co-Culture with Human Macrophages

Many approaches can be used to blockade overexpressed CD47 in solid tumors and AML, including the use of monoclonal antibodies.^3,4^ However, the use of CD47_siRNA and effective nanocarriers to suppress the production of CD47 protein is a powerful anticancer tool to study the dynamic relationship between CD47 and CRT-induced phagocytosis in multiple types of human cancers in co-culture. Different GO formulations were used as nanocarriers to carry siRNA inside many types of cancer cells.^27,45−47^ In a previous work, we have shown the use of GO flakes modified with PEG and PAMAM to carry CD47_siRNA inside AML cells. The knockdown efficiency of CD47 protein was 65–72% in AML cells compared to the levels of un-transfected cells.^29^ Due to its efficient delivery of CD47_siRNA to AML cells, the GO-PEG-PAMAM formulation with a size of roughly 100 nm (small-GO-PEG-PAMAM) was used in this study to carry siRNA into multiple cancer cells with a human macrophage co-culture model. The characterization of small-GO-PEG-PAMAM was previously published, and details can be found in the Supporting Information.

Human macrophages were differentiated from human monocytes by chemical modification using phorbol 12-myristate 13-acetate (PMA) as previously described.^48^ Cells showed increased adherence and macrophage-like morphology after PMA treatment. Moreover, macrophage markers such as CD11b and CD14 showed increased levels in PMA-treated cells compared to the non-treated control by flow cytometry (Figure S1A–C). Differentiated human macrophages were used in a co-culture model to investigate the elimination of cancer cells throughout the study.

The co-culture model used here was previously established for the selective elimination of AML cells by lipopolysaccharide (LPS)-stimulated macrophages.^9^ Results showed simultaneous CD47 downregulation and upregulation of CRT upon LPS treatment. Moreover, AML cells, but not normal cells, were eliminated by stimulated macrophages.^9^ Here, in the current study, our hypothesis states that the knockdown of CD47 (other than LPS-stimulated macrophages) in multiple cancer cells could increase their CRT levels and result in their elimination by macrophages in co-culture. Therefore, CD47 silencing by using siRNA and small-GO-PEG-PAMAM as a nanocarrier to cancer cells in co-culture could be compared to the LPS-stimulated phagocytosis in previous studies of ours and others.^5,9^

**Figure 1:**
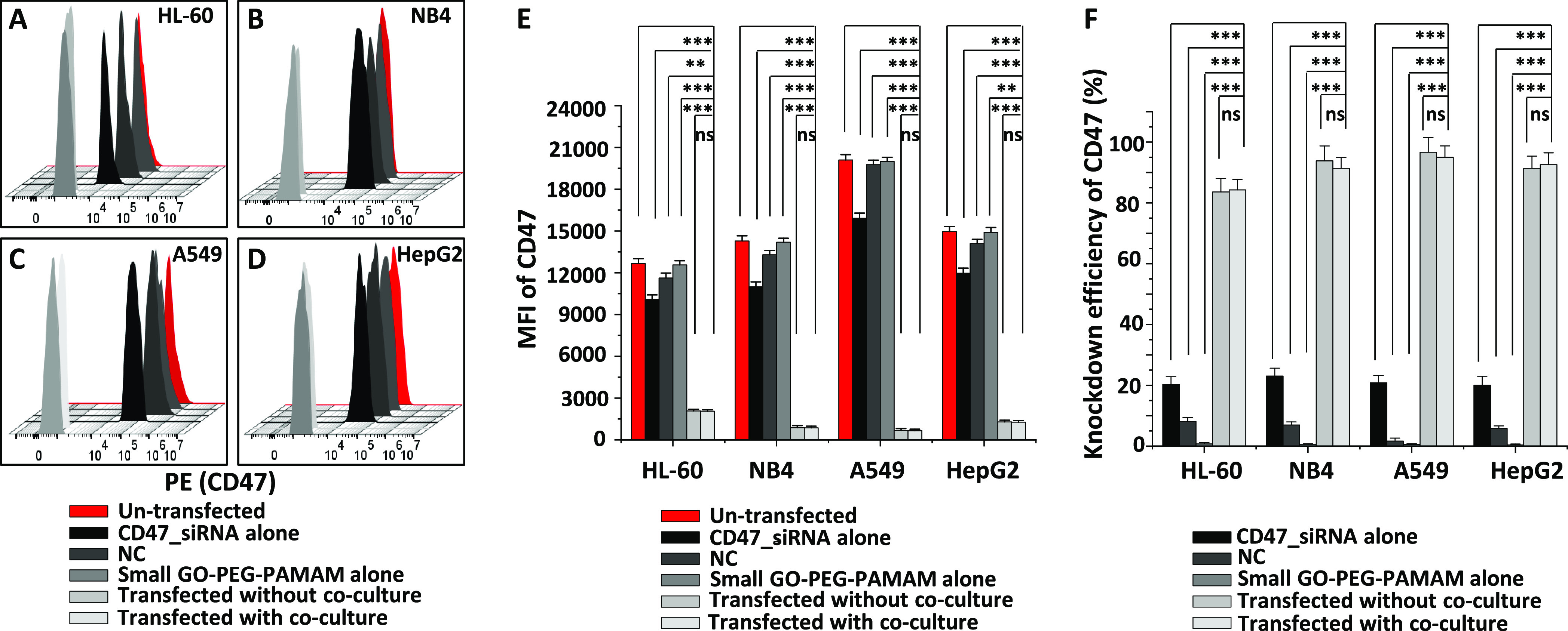
Knockdown of CD47 using small-GO-PEG-PAMAM in multiple cancer cell lines with and without co-culture with human macrophages. (A–D) Overlaid flow cytometric histograms representing the levels of PE-labeled CD47 protein before and after knockdown of CD47 in HL-60 (A), NB4 (B), A549 (C), and HepG2 (D). (E) Mean fluorescence intensity (MFI) of PE-labeled CD47 in all cell lines used. (F) Knockdown efficiency (%) of CD47 in all cell lines used. Values in the graphs are shown as the mean ± SEM of three trials of duplicate samples (*n* = 6). The statistical analysis of the MFI was determined by one-way ANOVA, measured on un-transfected, CD47_siRNA only, negative control (NC), and small-GO-PEG-PAMAM only samples and samples transfected with and without co-culture in each cell in panel (E) and CD47 knockdown efficiency in panel (F). **P < 0.01 and ***P < 0.001; “ns”, not significant.

To test our hypothesis, human HL-60, NB4, A549, and HepG2 cells were co-cultured with differentiated human macrophages through Transwell membrane inserts. CD47_siRNA was conjugated to small-GO-PEG-PAMAM by allowing them to interact at room temperature (RT) for 30 min. The negatively charged siRNA creates an electrostatic interaction with the positively charged dendrimers (PAMAM) on the surface of small-GO-PEG-PAMAM, resulting in a CD47_siRNA/small-GO-PEG-PAMAM complex. Then, the complex was transfected for 48 h in the above cancer cells in co-culture at a final ratio of 1:1 (final concentration of 0.25 μg/mL of both CD47_siRNA and small-GO-PEG-PAMAM). Small-GO-PEG-PAMAM (0.25 μg/mL) was added to human macrophages as a stimulus. The knockdown efficiency results of AML cells (Figure 1A,B,E,F) showed lower values (84 and 91%, respectively) than the adherent A549 (the highest knockdown efficiency of 95%, Figure 1C,E,F) and HepG2 cells (93%, Figure 1D–F). The knockdown efficiency for all cell lines was significantly higher than that for un-transfected cells or transfected cells using the negative control (5–8%) conjugated to small-GO-PEG-PAMAM (Figure 1F). It is worth noting that the knockdown efficiency of CD47_siRNA alone was an average of 20% in all cancer cells (Figure 1F). It is obvious that small-GO-PEG-PAMAM as a nanocarrier has significantly improved the knockdown of CD47 in all cancer cells. Moreover, the knockdown efficiency reported here for HL-60 and NB4 is higher than the one we obtained previously (61 and 71%, respectively) using the same GO carrier formulation.^29^ The possible reason is due to the improved siRNA sequence and the time of transfection. CD47_siRNA used in this study has 27 duplex RNA bases instead of the traditional 21 bases, which we used in the previous study. This has increased the potency of siRNA compared to the traditional 21 bases.^49^ In addition, a transfection time of 48 h was used here, which is better for the protein phenotypic responses.^50^ Transfection of CD47_siRNA using small-GO-PEG-PAMAM as a carrier in the same cancer cells without co-culture showed very similar knockdown efficiencies to the ones obtained with co-culture (Figure 1F), indicating that co-culture has no effect on the knockdown of CD47.

The cytotoxicity of small-GO-PEG-PAMAM was investigated *in vitro* at different concentrations on AML, A549, and HepG2 cells.^29^ Moreover, human macrophages were evaluated for their viability at 0.25 μg/mL of small-GO-PEG-PAMAM. Results showed a viability of more than 90% in all cell lines, as well as in macrophages, indicating the safe use of small-GO-PEG-PAMAM as a nanocarrier (Figure S2A,B).

The knockdown efficiencies obtained here using small-GO-PEG-PAMAM as a nanocarrier were higher than the ones reported using the same functional groups modified with GO targeting different proteins. For example, covalently modified GO with PEG and PAMAM was used to deliver siRNA to triple negative breast cancer cells *in vitro.*(51) The knockdown efficiency of the targeted protein was 61%. Other GO derivatives were reported by carrying siRNA to breast and ovarian cancer cells *in vitro* with lower knockdown efficiencies than the ones reported here.^45,47^ Our results suggested the high efficacy of small-GO-PEG-PAMAM to carry siRNA inside cancer cells *in vitro*.

### Effect of CD47 Knockdown Using Small-GO-PEG-PAMAM as a Nanocarrier on CRT Levels and Elimination of Multiple Cancer Cells in Co-Culture

**Figure 2:**
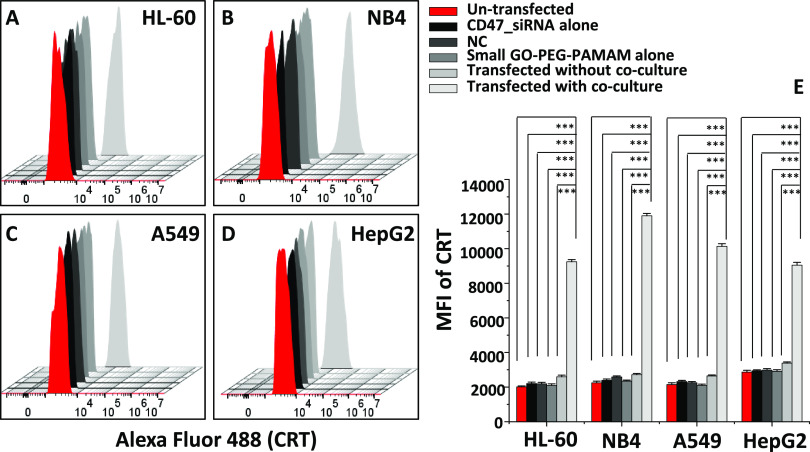
Calreticulin levels after CD47_siRNA transfection using small-GO-PEG-PAMAM in multiple cancer cell lines. (A–D) Overlaid flow cytometric histograms representing the levels of Alexa Fluor 488-labeled CRT protein before and after transfection of CD47_siRNA in HL-60 (A), NB4 (B), A549 (C), and HepG2 (D). (E) Mean fluorescence intensity (MFI) for Alexa Fluor 488 CRT in all cell lines. Values in the graphs are shown as the mean ± SEM of three trials of duplicate samples (*n* = 6). The statistical analysis of the MFI of the transfected sample with co-culture compared to those of the un-transfected, CD47_siRNA, negative control, and small-GO-PEG-PAMAM only samples and the sample transfected without co-culture in each cell line in panel (E) was determined by one-way ANOVA. ***P < 0.001.

CRT is a known pro-phagocytic signal on human cancer cells and is required for anti-CD47 antibody-mediated phagocytosis.^5,10^ In our study, the knockdown of CD47 by using CD47_siRNA conjugated to small-GO-PEG-PAMAM in co-culture with human macrophages supported these findings. Our results showed the highest levels of CRT on NB4 cells followed by A549, HL-60, and HepG2 cells after transfection of CD47_siRNA in co-culture (Figure 2A–D). This increase was significant (*P* < 0.01) compared to un-transfected cells (Figure 2E). However, when the transfection was carried out without co-culture, a slight increase in CRT level was seen, and it was not significant (*P* > 0.05) compared to the levels in un-transfected cells (Figure 2E).

**Figure 3:**
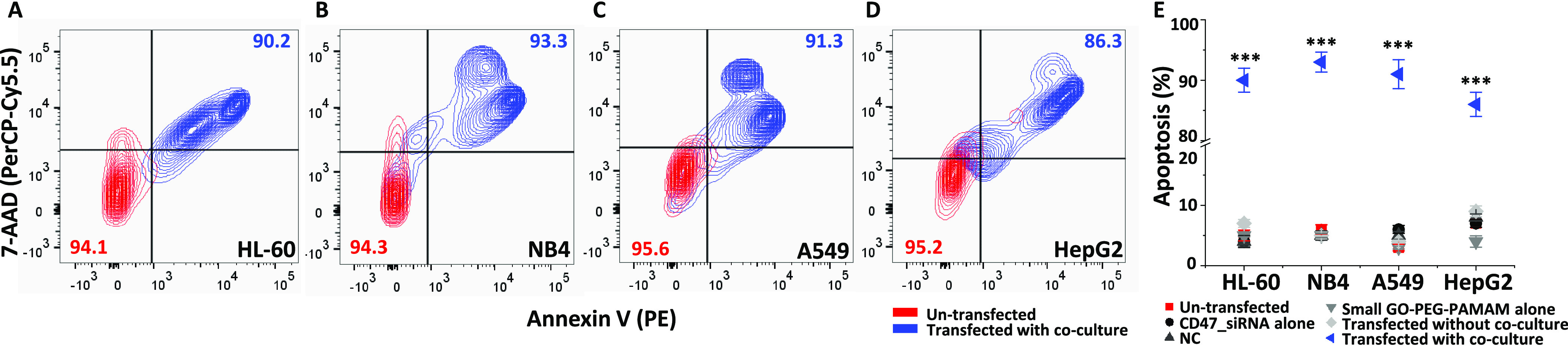
Elimination of multiple types of cancer cells by human macrophages in co-culture after CD47_siRNA transfection using small-GO-PEG-PAMAM as a nanocarrier. (A–D) Overlaid contour plots representing un-transfected and transfected samples with co-culture populations of cancer cells. Each plot shows the apoptosis of HL-60 (A), NB4 (B), A549 (C), and HepG2 (D) cancer cells. The bottom left quadrant specifies viable cells with intact membranes that are Annexin V- and 7-AAD-double-negative. The top left quadrant denotes necrotic cells that are 7AAD-positive and Annexin V-negative. The top right quadrant includes late apoptotic cells that are Annexin V- and 7AAD-double-positive. The right bottom quadrant designates early apoptotic cells that are Annexin V-positive but 7-AAD-negative. (E) Apoptosis summary (%) for each cell line. Values in the graph (E) are shown as the mean ± SEM of three trials of duplicate samples (*n* = 6). The statistical analysis of the transfected sample with co-culture compared to the un-transfected, CD47_siRNA, negative control, and small-GO-PEG-PAMAM only samples and the sample transfected without co-culture in each cell line in panel (E) was determined by one-way ANOVA. ***P < 0.001.

When in co-culture, the knockdown of CD47 increased the levels of CRT on cancer cells significantly, resulting in their elimination by macrophages. Results showed an apoptosis value of 93% for NB4 cells, followed by 91, 90, and 86% for A549, HL-60, and HepG2 cells, respectively (Figure 3A–E). Interestingly, in our results, there is a correlation between CRT levels and apoptosis by macrophages (Figure 2E and Figure 3E). This indicated that the presence of CRT might be required for apoptosis in transfected cells. Cancer cells without co-culture exhibit 4–9% apoptosis only (Figure 3E) despite the slight increase (∼10%, Figure 2E) in CRT after transfection. Our results suggest that subsequent elimination of cancer cells only takes place when the cancer cells were co-cultured with macrophages and CD47 was silenced. More investigation into the CRT levels and phagocytosis is studied with a direct co-culture model and discussed in following sections.

Few studies reported the relationship between CD47 and CRT in cancer cells when CD47 is downregulated *in vitro.*(52) From our results, the knockdown of CD47 in co-culture and the significant increase in CRT level in cancer cells took place simultaneously. These results matched our previous report when LPS was used to downregulate CD47 in co-culture,^29^ suggesting that small-GO-PEG-PAMAM/CD47_siRNA and LPS might be acting similarly in downregulating CD47 and upregulating CRT in multiple types of cancer cells in co-culture.

It has been reported that blocking CD47 levels on the surface of cancer cells led to increased secretion of cytokines by macrophages to stimulate phagocytosis.^53,54^ Herein, we used CD47_siRNA conjugated to small-GO-PEG-PAMAM to knock down the levels of CD47 in all cancer cell lines used. As a result, high knockdown efficiency was seen in all cancer cell lines. We also found that the knockdown of CD47 resulted in significant production of IL-6, TNF-α, IL-1β, and IL-8 in the supernatant of the media in co-culture compared to the control (un-transfected cancer cells with co-culture) (Table 1). IL-8, IL-6, and TNF-α were present in the supernatant with a more than 65-fold increase compared to the control. IL-1β was over 40-fold higher than the control (Table 1). The increase in production of such cytokines indicates that M1 macrophages are present, and therefore, their main role was elimination of cancer cells.^55^ These findings supported the finding that CD47 knockdown using CD47_siRNA and small-GO-PEG-PAMAM as nanocarriers in co-culture increased levels of CRT on cancer cells to flag them for elimination by M1 macrophages, which validates our hypothesis. Moreover, results of cytokine levels were similar to those obtained when LPS was used to stimulate macrophages in co-culture.^9^ This is the second evidence to prove the similarity of small-GO-PEG-PAMAM/CD47_siRNA and LPS in downregulation of CD47 and elimination of cancer cells in co-culture.

**Table 1:** Cytokine Levels Secreted by Human Macrophages after CD47 Knockdown Using Small-GO-PEG-PAMAM as a Nanocarrier in Co-Culture

	IL-6	TNF-α	IL-1β	IL-8	IL-10	IL-2	IFN-γ
level of cytokine (pg/mL)	8968	8736	5956	10,359	1363	256	298
fold increase of cytokines relative to control	71	69	46	82	12	1	1
***P < 0.001;*P < 0.05; “ns”, not significant	***	***	***	***	*	ns	ns

### Knockdown of CD47 and Changes of CRT Levels Using Lipid Nanoparticles and Modified Boron Nitride Nanotubes as Nanocarriers

To study whether CRT levels are always elevated when CD47 is knocked down, and if this increase is required for cancer cell elimination in co-culture, we investigated the role of other nanocarriers when conjugated to CD47_siRNA. LNPs and BNPs were used to carry CD47_siRNA inside HL-60, NB4, A549, and HepG2 cancer cells *in vitro*.

**Figure 4:**
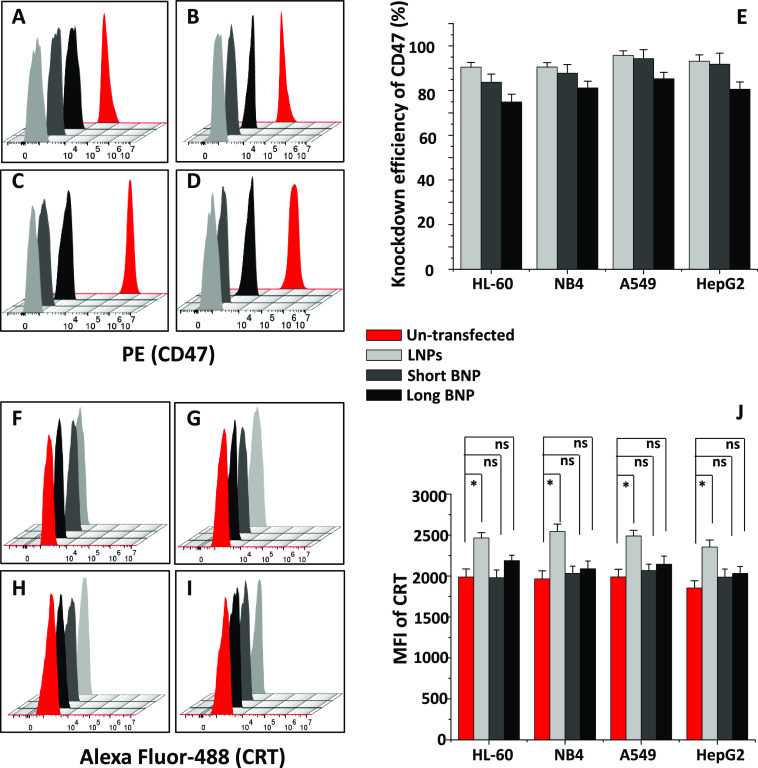
Flow cytometry analysis of CD47 and CRT levels after transfection of CD47_siRNA using LNPs and short and long BNPs as nanocarriers in co-culture with human macrophages. (A–D, F–I) Flow cytometry histograms showing the mean fluorescence intensity (MFI) of PE-labeled CD47 and Alexa Fluor-488-labeled CRT proteins, respectively, in HL-60 (A, F), NB4 (B, G), A549 (C, H), and HepG2 (D, I) cells. (E) Knockdown efficiency of CD47 in all cancer cell lines after transfection in co-culture with human macrophages. (J) MFI of Alexa Fluor-488 CRT in all cancer cell lines used. The statistical analysis of the MFI of the transfected sample with co-culture for each of the nanocarriers compared to that of the un-transfected sample in panel (J) was determined by one-way ANOVA. *P < 0.05; “ns”, not significant.

LNPs represent one of the most advanced systems for siRNA delivery *in vitro* and *in vivo.*(56,57) CD47_siRNA encapsulated in LNPs was delivered to HL-60, NB4, A549, and HepG2 cells, and results showed values of CD47 knockdown efficiency above 90% in co-culture in all cell lines used (Figure 4A–D). Lung and liver cancer cell lines showed 96 and 93% knockdown efficiencies, respectively, whereas AML suspension cells showed 91% for both HL-60 and NB4 (Figure 4E). When using the negative control of siRNA with LNPs, and when CD47_siRNA was transfected alone (without carriers), low knockdown efficiency values were found, which were similar to the ones obtained by using small-GO-PEG-PAMAM (Figure S3A,B). Moreover, transfection of CD47_siRNA using LNPs with and without co-culture showed similar CD47 knockdown efficiency (Figure S3A,B). However, when using LNPs, the knockdown of CD47 in HL-60 improved by 8% compared to small-GO-PEG-PAMAM (Figures 1F and 4E). It is known that using the DLin-MC3-DMA ionizable amino lipid enhances the potency and improves the cellular uptake of siRNA, especially in hard-to-transfect cells such as suspension cells.^58,59^ Therefore, a high knockdown of CD47 in HL-60 was expected.

Some studies reported the use of the same type of LNP with a similar knockdown efficiency of different target proteins in leukemia cells^58^ and hepatocytes.^60^ Others used different types of LNPs conjugated with CD47_siRNA in lung and colon cancers. These studies reported knockdown efficiency values of CD47 between 65 and 80%.^61,62^ The cytotoxicity of LNPs used here was studied on all cell lines. Results showed high viability (more than 90%) at the concentration used here in cancer and normal cells (Figure S4).

Short BNNTs (∼200 nm) were produced by sonicating the BNNT dispersion (sizes are typically between 500 nm and a few micrometers) for 5 h. Short and long BNNTs were modified with poly(3-methoxy tetraethyl methyl theophany) to obtain a stable dispersion in water and cell media, with a reasonable cellular uptake (Figure S5). These modified BNNTs are named as BNP (BNNT-polymer). Atomic force microscopy height imaging was used to assess the sizes of BNPs (Figure S6A,B). When using the short BNP, results showed that CD47 knockdown efficiency values were similar to those obtained when using small-GO-PEG-PAMAM and LNPs in A549 (94%), HepG2 (92%), and NB4 (89%) cells (Figure 4A–E). However, knockdown in HL-60 (84%) was lower than the one obtained from LNPs and similar to the small-GO-PEG-PAMAM. When using long BNP as a nanocarrier, however, CD47 knockdown efficiency values were the lowest in all cell lines (Figure 4E), with 85% found in A549 cells, 73% in HL-60 cells, and 80% in both NB4 and HepG2 cells (Figure 4A–E). The low knockdown of CD47 when using long BNP could indicate the low efficiency of cellular uptake of CD47_siRNA conjugated to long BNP, likely due to the longer tube length (some are longer than 2–3 μm and entangled with each other, Figure S6). Transfection of CD47_siRNA alone, as well as without co-culture for both BNP formulations, obtained similar results compared to the rest of the nanocarriers (Figure S6C,D). Short and long BNPs at the concentrations used here showed no significant cytotoxicity in any cancer cells (Figure S6E).

Commercially available lipofectamine RNAiMAX was used to carry CD47_siRNA into all cell lines to compare with the selected nanocarriers used here. Results showed knockdown efficiencies of 71, 64, 55, and 46% in A549, HepG2, NB4, and HL-60 cells, respectively (Figure S7A–F). No difference of the knockdown efficiency was observed in cells with or without co-culture. The values reported here were still lower than the ones obtained when using each of the nanocarriers mentioned above. This indicates the effective role of the selected nanocarriers compared to the standard lipids in carrying CD47_siRNA in different types of cancer cells.

CRT levels have slightly increased in all cell lines used after transfection of CD47_siRNA using LNPs as a nanocarrier in co-culture (Figure 4F–J). However, the increase in CRT level with co-culture in the case of small-GO-PEG-PAMAM was 5-fold higher after transfection in NB4 cells and around 4-fold higher in HL-60, A549, and HepG2 cells (Figure 2E). This could indicate a vital role of CRT in apoptosis when small-GO-PEG-PAMAM/CD47-siRNA was used. The increase in CRT level after transfection without co-culture and for CD47_siRNA alone was similar to that for the un-transfected sample (Figure S8A). When using both BNP nanocarriers, the increase in CRT level after transfection of CD47 in co-culture and without co-culture was very similar to that for un-transfected cells (not significant (*P* < 0.05)) (Figure 4J and Figure S8B,C).

**Figure 5:**
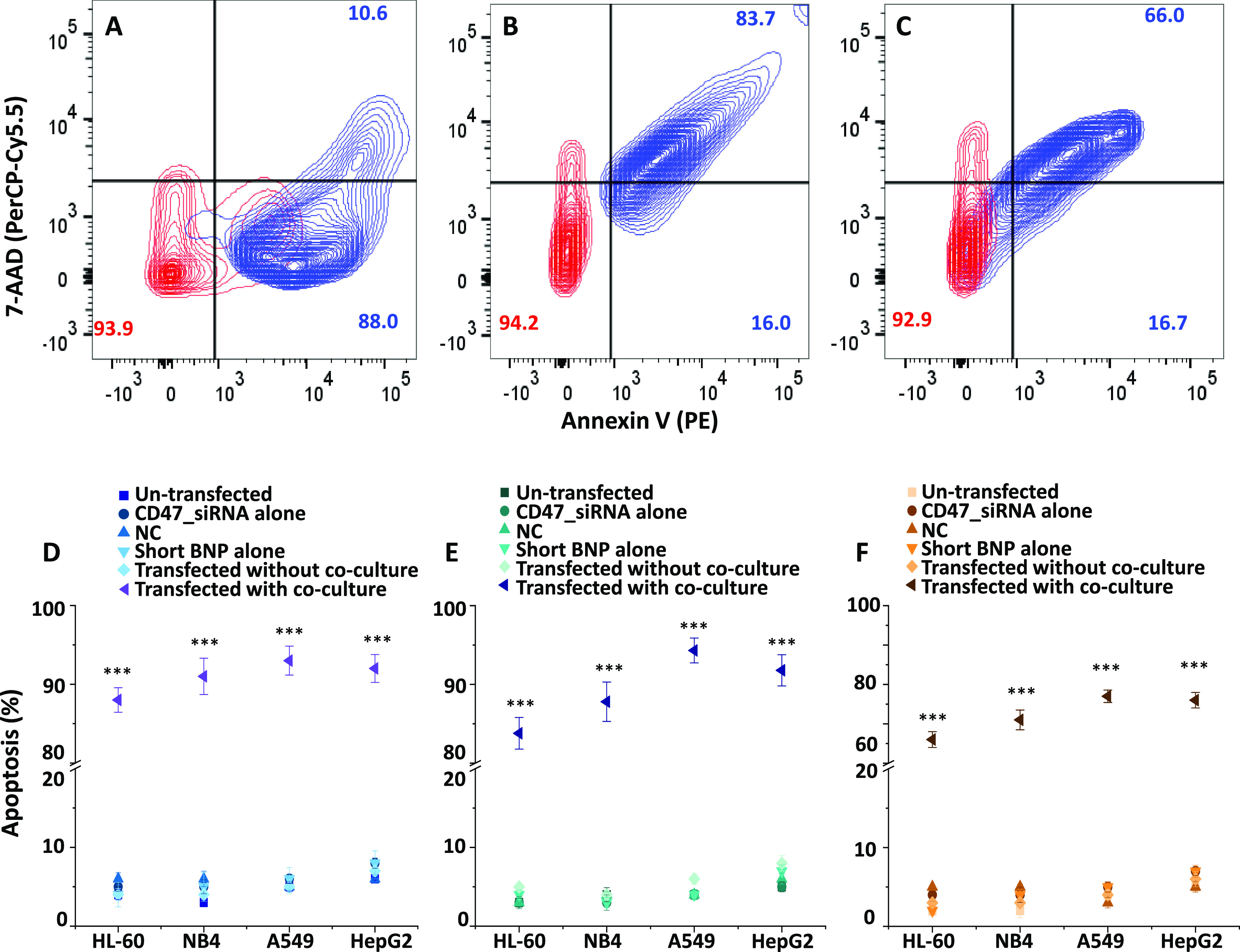
Elimination of cancer cells by human macrophages in co-culture after CD47_siRNA transfection using LNPs and short and long BNPs as nanocarriers. (A–C) Representative overlaid contour plots for HL-60 cells with LNPs (A), short BNP (B), and long BNP (C), representing populations of un-transfected and transfected cells in co-culture. The bottom left quadrants specify viable cells with intact membranes that are Annexin V- and 7-AAD-double-negative. The top left quadrants denote necrotic cells that are 7-AAD-positive and Annexin V-negative. The top right quadrants include late apoptotic cells that are Annexin V- and 7-AAD-double-positive. The right bottom quadrants designate early apoptotic cells that are Annexin V-positive but 7-AAD-negative. (D–F) Apoptosis summary (%) for all cell lines used after transfection of CD47_siRNA conjugated to LNPs (D), short BNP (E), and long BNP (F). Values in the graphs represent early and late apoptosis, shown as the mean ± SEM of three trials of duplicate samples (*n* = 6). The statistical analysis of the transfected sample with co-culture compared to the un-transfected, CD47_siRNA, and negative control samples, samples with each of the nanocarriers alone, and the sample transfected without co-culture in each cell line was determined by one-way ANOVA. ***P < 0.001.

HL-60 cells showed apoptosis levels of 88 and 84% in LNPs and short BNP, respectively (Figure 5A,B). Apoptosis levels of around 90% were found in adherent solid tumor cells (A549 and HepG2) with co-culture when using LNPs (Figure 5D) and short BNP (Figure 5E) as nanocarriers. Apoptosis in NB4 cells was slightly higher when using LNPs (Figure 5D) than short BNP (Figure 5E). When looking at the corresponding values using long BNP, apoptosis registered the lowest values of 66% for HL-60 cells, 71% for NB4 cells, and 77% for both A549 and HepG2 cells (Figure 5F). These low values were the result of the low knockdown of CD47 obtained by using long BNP as a carrier.

Unlike small-GO-PEG-PAMAM/CD47_siRNA, there was no correlation between CRT levels and apoptosis for cancer cells transfected with CD47_siRNA encapsulated inside the LNPs or conjugated with both BNPs in co-culture. Instead, apoptosis results seemed to be correlated with the knockdown efficiencies of CD47 regardless of CRT levels. We observed that the higher the knockdown of CD47 in cancer cells, the higher the percentage of apoptosis.

### Investigating the Phagocytosis of Cancer Cells when Blocking Surface CRT and the Knockdown of CD47 by All the Nanocarriers

**Figure 6:**
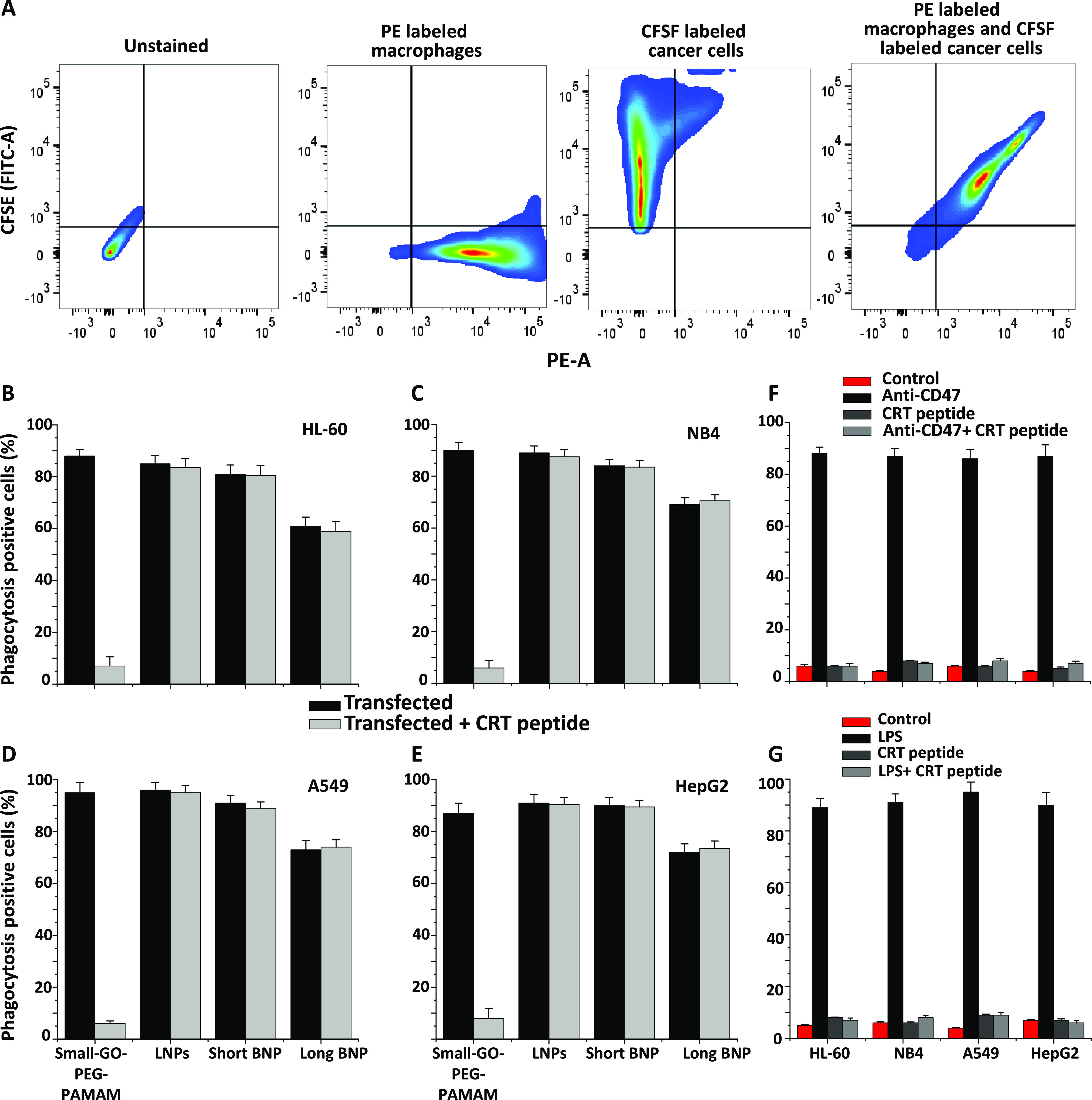
Phagocytosis assay in multiple types of cancer cells with direct co-culture with human macrophages after transfection of CD47_siRNA conjugated to each of the nanocarriers and CRT blocking *in vitro*. (A) Experimental design showing the labeling and gating strategy of all cancer cells and macrophages. Unstained cells appear on the lower left of the scatter dot plot, whereas PE-labeled macrophages and CFSE-labeled cancer cells appear on the lower right and upper left, respectively. Phagocytosis-positive cancer cells are shown with labeled macrophages together on the upper right of the dot plot. (B–E) Phagocytosis-positive cells (%) after transfection of CD47_siRNA conjugated to each of the nanocarriers and after transfection and CRT blocking on cancer cells by the CRT peptide (4 μg/mL) at the same time in HL-60 (B), NB4 (C), A549 (D), and HepG2 (E) cells. (F, G) Phagocytosis-positive cells (%) in all cancer cell lines after blocking of CD47 by the B6H12 CD47 antibody ((F) 10 μM/mL) or after treatment of LPS (100 ng/mL), CRT blocking by the CRT peptide, and blocking CD47 with B6H12 and CRT with the CRT peptide together. Control cells are cancer cells that had not been treated with the CD47 antibody, LPS, or CRT blocking peptide.

In the literature, the unmodified BNNTs were used to deliver different payloads inside several types of cancers.^36,63−65^ The CRT levels obtained before and after the downregulation of CD47 using siRNA silencing via BNP carrier formulations have provided insight into exploring the role of CRT in phagocytosis using different nanocarriers. Therefore, more studies have followed to focus on CRT level measurements. To investigate whether CRT protein is the only dominant signal for phagocytosis by macrophages, HL-60, NB4, A549, and HepG2 cancer cells were each co-cultured directly (without a Transwell membrane dividing the two cells in the co-culture chamber) with human macrophages (Figure 6). Direct co-culture was performed to measure the actual phagocytosis between cancer cells and human macrophages after direct contact in the same well in the cell culture plate. Knockdown efficiency evaluations of CD47 were performed by transfection of CD47_siRNA via four nanocarriers. The CD47 blocking antibody (B6H12, 10 μg/mL) was used to block CD47 in all cancer cells. LPS was used to downregulate CD47 as previously described.^9^ The CRT blocking peptide (4 μg/mL) was used to block surface CRT. Cancer cells were either transfected with CD47_siRNA via each of the four nanocarriers only or transfected in addition to the CRT blocking peptide at the same time to block surface CRT. Moreover, cancer cells were either treated with B6H12 or LPS alone, or treated with B6H12 or LPS and CRT blocking peptide at the same time. After 48 h of incubation in direct co-culture, the phagocytosed cancer cells (which are double-positive in both FITC (cancer cells) and PE (human macrophages) filter channels) were measured by flow cytometry (Figure 6A).

When small-GO-PEG-PAMAM was used as a carrier to knock down CD47, phagocytosis took place in all cell lines used (Figure 6B–E). However, when CRT was blocked using the CRT blocking peptide during the transfection, no phagocytosis was seen in any of the cell lines (Figure 6B–E). This indicated the requirement of CRT on the surface of cancer cells to be phagocytosed when they are transfected with CD47_siRNA conjugated to small-GO-PEG-PAMAM. When LNPs and short and long BNP nanocarriers were used to knock down CD47 with or without CRT blocking, phagocytosis happened in all cell lines (Figure 6B–E). This showed that the presence of CRT was not required for the phagocytosis and there might be other pathways involved. This is consistent with what has been observed in co-culture (with the Transwell membrane experiments) without CRT blocking (Figure 4F–I).

All nanocarriers were compared to the commercially available B6H12 anti-CD47 antibody, which was used to block CD47 in all cancer cell lines in co-culture. Results showed that cancer cells were phagocytosed by macrophages only when CRT was not blocked (Figure 6F). When the CRT peptide was used for blocking surface CRT alone with or without anti-CD47 antibody blocking, no phagocytosis took place in all cell lines (Figure 6F), indicating that an elevated CRT level is required for phagocytosis. In our previous study, LPS was used to downregulate CD47 in AML cancer cells. As a result, CD47 downregulation and CRT elevation were observed at the same time.^9^ However, no investigation took place on whether CRT was required for the phagocytosis of cancer cells. Herein, surface CRT in cancer cells was blocked along with LPS treatment (Figure 6G). Results showed that CRT was indeed a requirement for cancer cells to be phagocytosed (Figure 6G). Un-transfected and untreated cells (controls) showed no phagocytosis when directly co-cultured with human macrophages when using B6H12 and LPS (Figure 6F,G).

These results were similar to the ones obtained when B6H12 was used. Moreover, it seems that LPS treatment and CRT blocking at the same time showed similar results to small-GO-PEG-PAMAM/CD47_siRNA here. Therefore, we can conclude that downregulating CD47 by using small-GO-PEG-PAMAM/CD47_siRNA and LPS involves the same pathway of phagocytosis, which is CRT-mediated phagocytosis.

CD47_siRNA transfection with the small-GO-PEG-PAMAM nanocarrier seems to follow the same path as the LPS-stimulated downregulation of CD47. Transfected cancer cells via LNPs and BNP nanocarriers with CD47_siRNA were CRT-independent for phagocytosis. Other pathways could be activated for phagocytosis in this case. The interaction of CRT on the surface of cancer cells with its receptor (low-density lipoprotein receptor) on the macrophages leads to the CRT-mediated phagocytosis of cancer cells.^5,66^ Blocking of this interaction by the CRT blocking peptide prevents phagocytosis by macrophages in co-culture. Some studies reported that CRT could be the dominant phagocytic trigger in multiple types of cancer cells when CD47 was blocked by the anti-CD47 antibody *in vitro*.^5^ However, other phagocytosis pathways independent of CRT have also been reported.^67^ Signaling lymphocytic activation molecule family-7 (SLAMF7) was found to be a pro-phagocytosis “eat me” signal on the surface of hematopoietic tumor cells. SLAMF7-mediated phagocytosis occurs in hematopoietic tumor cells upon the blockade of CD47 on their surfaces.^67^ Others reported that the activation of phagocytosis-activating receptors such as Fc IgG (FcγRs) in lymphoma and leukemia patients increases the phagocytosis of cancer cells.^68^

Our results indicate that CRT was dominant for phagocytosis when small-GO-PEG-PAMAM/CD47_siRNA was used to downregulate CD47 in AML, A549, and HepG2 cells *in vitro* in co-culture. However, CRT seemed to be less affected when using LNPs and short or long BNP as nanocarriers for CD47 knockdown in the same co-culture model. Phagocytosis still took place in cancer cells when these carriers were used, indicating that the elevated CRT level is unlikely the dominant pro-phagocytic signal in those cases using LNPs and BNPs as carriers.

Graphene oxide and boron nitride nanotubes were used successfully to carry CD47_siRNA inside different types of cancer cells. Both of these nanocarriers were safe to use *in vitro* at the concentration used here. However, the *in vivo* toxicity of GO and BNNT is always a considered factor for clinical application due to their poor degradation levels. To increase the use of these nanocarriers in clinical applications *in vivo*, biodegradable polymers, including synthetic polymers such as poly(ethylene glycol)-*block*-poly(lactide) copolymer (PEG-*b*-PLA), or natural polymers such as albumin, gelatin, collagen, and chitosan, could be used as coating layers^69−71^ to prolong the blood circulation and achieve sufficient target accumulation. Once triggered by stimuli or another treatment, the biodegradable polymers could be dissociated from GOs and BNNTs, allowing the rapid clearance of these nanomaterials from organs such as livers and spleens. Alternatively, new designs with different crystalline structures of these materials could also provide opportunities for using these particles as nanocarriers in developing novel therapies.^72−74^

## Conclusions

In our study, modified graphene oxide, boron nitride nanotubes, and lipid nanoparticle formulations as nanocarriers were used successfully for the delivery of CD47_siRNA in AML, lung, and liver cancer cells in co-culture with human macrophages *in vitro*. High knockdown efficiency values were obtained as a result of transfection of CD47_siRNA for all nanocarriers used. The effect of the knockdown of CD47 on CRT levels and phagocytosis in cancer cells was investigated. Small-GO-PEG-PAMAM as nanocarriers resulted in a high CD47 knockdown efficiency and significant increase in CRT level in all cancer cells. The presence of CRT on cancer cells was required for phagocytosis by macrophages in co-culture. These results confirm our previous work when LPS was used to stimulate macrophages in co-culture with AML cells. Downregulation of CD47 and upregulation of CRT simultaneously took place in cancer cells and allowed for the selective elimination of cancer cells by macrophages in co-culture.^9^ Small-GO-PEG-PAMAM and LPS seem to act as the same pathway for cancer cell elimination. However, when using LNPs and two BNP carrier formulations, CRT was independent of phagocytosis, indicating the involvement of other pathways in the cancer cell elimination process. Results obtained here highlight the dynamic relationship between CD47 and CRT in human cancers. Moreover, they support the development of anti-CD47 therapies in multiple cancer types by the potential use of the above nanocarriers as a platform to screen different drugs *in vitro*.

## Materials and Methods

### Preparation of Nanocarriers

CD47_siRNA and all reagents needed for transfection were purchased from Integrated DNA Technologies (IDT, USA). The CD47_siRNA duplex sequence used for transfection in all cell lines used in this study is 5′-rGrCrArArCrArArCrCrUrUrUrCrCrArGrCrUrArCrUrUrUTG-3′ and 5′-rCrArArArArGrUrArGrCrUrGrGrArArArGrGrUrUrGrUrUrGrCrArG-3′.

The negative control was provided as a “universal negative control”, not a scrambled sequence of the above. The actual sequence was not revealed by the provider (IDT, USA).

Synthesis, purification, and modification of GO have followed previously developed methods.^29^ Verification of GO modification was previously carried out, and the loading of siRNA was confirmed by measuring the size and surface charge changes by dynamic light scattering^29^ (see the Supporting Information for more details). Lipid nanoparticles (LNPs) containing the DLin-MC3-DMA ionizable amino lipid, cholesterol, distearoylphosphatidylcholine (DSPC), and 1,2-dimyristoyl-*rac*-glycero-3-methoxypolyethylene glycol-2000 (DMG-PEG2000) lipid at a molar ratio of 50/38.5/10/1.5 were combined (in ethanol and acetate buffer, pH 6) with CD47_siRNA and negative control of CD47_siRNA in two separate formulations using a commercial microfluidic mixer (NanoAssemblr Benchtop). Dynamic light scattering (DLS) followed by polydispersity (PDI) characterization and zeta potential (mV) measurement was performed in Prof. Walker’s Lab (University of Toronto, Ontario, Canada). LNPs with CD47_siRNA and the negative control (at an N/P ratio of 6:1) showed 95 and 116 nm, with PDI values of 0.17 and 0.18 at −3.0 and −2.7 mV for each formulation, respectively. Synthesis,^75^ purification^76,77^ and polymer modification of BNNTs^41,42^ followed the approaches developed previously. Briefly, the BNNTs were synthesized by the hydrogen-assisted boron nitride nanotube synthesis (HABS) process. Small diameter (∼5 nm) BNNTs were produced in high yield directly from hexagonal boron nitride (h-BN).^75^ The as-produced BNNT material was purified by chlorine etching at 950 °C.^76^ This gas-phase purification method removes boron impurities and can also remove some BN derivatives. Purified BNNTs in acetone (0.1 mg/mL) were noncovalently modified with the conjugated polymer poly(3-methoxy tetraethoxy methyl thiophene)^42^ and redispersed in water to make a 0.5 mg/mL BNNT-polymer (long BNP) dispersion. The dispersion was bath-sonicated for 300 min to obtain the short BNP dispersion. Ten milliliters of CD47_siRNA (0.5 μg/mL) was mixed with 10 mL BNP dispersions (0.5 μg/mL, in transfection media) at RT for 30 min to form the BNP nanocarrier formulations. Ultraviolet–visible (UV–Vis) absorption measurements (Cary 5000 spectrophotometer, Agilent) using 10 mm path length quartz cuvettes (Figure S5) were carried out. Atomic force microscopy (AFM) topography imaging was performed on a MultiMode with a NanoScope V controller (Bruker Nano Surfaces Division, Santa Barbara, CA, USA), in Peak Force QNM mode, to verify the size of the BNPs (Figure S6). The peak force with which the tip taps the sample surface was always kept at the lowest stable imaging level of 200–400 pN. Silicon nitride ScanAsyst-Air AFM probes (Bruker AFM Probes, Camarillo, CA, USA) were used in all peak force feedback measurements.

### Cell Lines and Cell Culture

A549 (lung carcinoma), HepG2 (liver carcinoma), HL-60, NB4 (acute myelocytic leukemia), and SC (human normal monocyte/macrophage) cells were purchased from American Type Culture Collection (ATCC, USA). All cell lines were cultured in Dulbecco’s modified Eagle’s medium (DMEM) and 10% fetal bovine serum (FBS), except for HL-60 and SC cells at 20% FBS in Iscove’s modified Dulbecco’s medium (IMDM). All cells were maintained at low passage numbers (<15) and cultured in a 5% CO_2_ and 95% humidified incubator at 37 °C.

### Transfection of CD47_siRNA Using Different Nanocarriers and Co-Culture Experiments

All cell lines mentioned above were plated at 2 × 10^5^ to 3 × 10^5^ cells per well in six-well tissue culture plates in reduced serum transfection media (Opti-MEM, ThermoFisher, Canada). CD47_siRNA and each of the nanocarriers were diluted in transfection media separately, and then each nanocarrier was added to the diluted CD47_siRNA to make the transfection mix-nanocarrier at a final ratio of 1:1 (at a final concentration of 0.25 μg/mL of both CD47_siRNA and each of the nanocarriers) and incubated for 30 min at RT (21 ± 2 °C). Then, the mix was added to each of the plated cell lines and allowed to incubate for 48 h in the incubator.

For co-culture experiments, SC cells were differentiated to human macrophages by adding phorbol 12-myristate 13-acetate (PMA) (Sigma, Canada), resulting in cells with increased adherence and loss of proliferative activity. Briefly, SC cells were plated (2 × 10^5^ to 3 × 10^5^ cells) in a six-well tissue culture plate, and then PMA (10 ng/mL) was added to the cells and incubated overnight at 37 °C. Cells were then washed with PBS, and fresh media were added. Macrophage surface markers (CD11b and CD14) were measured by anti-CD14 and (BD-Biosciences, Canada) anti-CD11b (BioLegend, USA) antibodies before and after SC differentiation. Differentiated SC cells were co-cultured with each of the cancer cell lines by 6.5 mm Transwell permeable, 0.40 μm pore polyester membrane supports (Sterlitech, USA). Then cancer cells were transfected with CD47_siRNA and each of the nanocarriers as described above, then they were incubated for 48 h in the CO_2_ incubator. SC macrophages were stimulated by the nanocarrier alone at concentration of 0.25 μg/mL.

Different controls were used to ensure that CD47_siRNA was delivered specifically to the target cells, including the delivery of CD47_siRNA alone, a universal negative control (NC) of siRNA, and the delivery of the nanocarrier without any siRNA.

### Flow Cytometry and Apoptosis Analysis with and without Co-Culture with Human Macrophages

AML cells were collected and adherent cancer cells were harvested by trypsin digestion. Then, all cells were washed twice with cold PBS and centrifuged at 400*g* for 5 min. Next, cells (1 × 10^6^) were resuspended in 100 μL of cell staining buffer (BioLegend, USA). Subsequently, all cancer cells were stained with PE-conjugated anti-human CD47 (BioLegend, USA) alone for cells without co-culture and with anti-human CD47 and Alexa Fluor 488-conjugated anti-human calreticulin (CRT) (Abcam, Canada) antibodies simultaneously for cells with co-culture. All stained cells were then incubated for 30 min in the dark at RT. Cells were washed twice with PBS, centrifuged at 400*g* for 5 min, and resuspended in 0.5 mL of cell staining buffer containing 1% formaldehyde. For apoptosis measurements, cells were harvested the same way mentioned above and stained for apoptosis using the PE-Annexin V apoptosis detection kit following the manufacturer’s instructions (BD Biosciences, Canada). All stained cells were analyzed by flow cytometry (CytoFLEX 5, Beckman Coulter, USA).

To determine the percentage of CD47 knockdown efficiency in all cancer cells used here, the following formula was used:
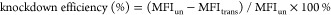
where MFI_un_ is the mean fluorescence intensity of un-transfected cells, and MFI_trans_ is the mean fluorescence intensity of cells transfected with a transfection mix-nanocarrier, cells transfected with CD47_siRNA alone, or cells transfected with each of the nanocarriers alone.

### Cytokine Measurements after Transfection of CD47_siRNA Using Small-GO-PEG-PAMAM

For measuring the levels of the cytokines IL-6, TNFα, IL-1β, IL-8, IL-10, IL-12, and IFN-γ, cell culture supernatants were collected from the human macrophage chambers after co-culture and transfection. The supernatants were stored at −80 °C and thawed to RT. They were centrifuged at 1500*g* for 10 min to exclude any cell debris during the collection process. A commercially available, Milliplex, human high-sensitivity T-cell magnetic bead panel kit (Millipore, USA) was used to measure the levels of the above cytokines in the supernatants, following the manufacturer’s instructions. The fluorescence intensity was detected using the MAGPIX system (Luminex, USA). The standard kit and cell samples were added in duplicate wells. Cytokine concentrations were calculated after the collection of standard curves and used at the protein level (pg/mL). The data were processed using Milliplex analyst software (version 5.1 Flex, VigeneTech, USA). Control supernatants were the ones obtained after macrophage stimulation with small-GO-PEG-PAMAM alone with co-culture and without transfection.

### Cytotoxicity Measurements for All Nanocarriers

The cytotoxicity of all the nanocarriers alone was evaluated in all cell lines used here at different concentrations (0, 0.25, 1, and 5 μg/mL) and incubated for 48 h in the CO_2_ incubator. Moreover, the cytotoxicity of the exact concentration (0.25 μg/mL) of each of the nanocarriers that was added to human macrophages was also evaluated. Briefly, the different concentrations mentioned above were diluted out of the stocks of each of the nanocarriers and added to complete growth media. Then, they were added to six-well tissue culture plates with 2 × 10^5^ to 3 × 10^5^ cells per well and then incubated for 48 h in the CO_2_ incubator. A PE-Annexin V apoptosis detection kit (BD-Biosciences, Canada), following flow cytometry analysis, was used to determine the viability percentage (obtained from flow cytometry PE scattered plots as the double-negative cell population) of cancer cells after treatment of all nanocarriers at the different concentrations, and the same analysis was applied for human macrophages.

### Phagocytosis Measurements and Direct Co-Culture

Cancer cells were stained with CellTracker stain carboxyfluorescein diacetate succinimidyl ester (CFSE) (ThermoFisher, Canada). Cancer cells were then washed (1 × 10^6^ at a time) with 1× PBS and centrifuged at 400*g* for 5 min. Then, cells were resuspended in 500 μL of 5 μM CFSE and allowed to incubate at RT for 20 min. To remove excess CFSE, cells were incubated with complete media containing serum and then centrifuged at 400*g* for 5 min. Cells then were washed again with 1× PBS and were ready for treatment and direct co-culture.

Stained cancer cells with CFSE were treated as follows: HL-60 and NB4 cells were transfected with CD47_siRNA and each of the four nanocarriers, blocked with the CRT blocking peptide (4 μg/mL, MBL International Corporation, USA), and added immediately to plated human macrophages in a six-well plate at a ratio of 2:1 (cancer cells to macrophages). For adherent cancers, stained CFSE cells were plated overnight to allow to adhere. Then, the next day, cells were transfected and treated as AML cells, and then human macrophages were added to the cancer cells with the same ratio as in AML. Treated cancer cells were allowed to incubate for 48 h in the CO_2_ incubator. After that, cells from direct co-culture were harvested and macrophages were stained for the CD11b antibody. Then, phagocytosis analysis was performed as follows: the cancer cells that are double-positive in both FITC (cancer cells) and PE (human macrophages) filter channels were considered as phagocytosed cells, and the percentage of phagocytosed cells was obtained from flow cytometry scatter dot plots after performing gating analysis (Figure 6A). Controls of unstained cells, CFSE-stained cancer cells only, and CD11b-stained macrophages only were prepared along with the phagocytosis analysis. The viability of all harvested cells was evaluated using trypan blue before flow cytometry analysis.

Cancer cells were treated with the B6H12 CD47 antibody (10 μM/mL, Bio Cell, USA) or LPS (100 ng/mL, Sigma, USA) and blocked with the CRT blocking peptide and CRT peptide only. Then, direct co-culture with human macrophages was applied as mentioned above for 48 h in the CO_2_ incubator. Control cells were not treated with either B6H12, LPS, or CRT blocking peptide.

## Supporting Information Available

Additional control experimental results on differentiation of human macrophages; viability of all nanocarriers at different concentrations; and characterization of the different nanocarriers (PDF)
