# Supplementary material for: Elimination of Cancer Cells in Co-Culture: Role of Different Nanocarriers in Regulation of CD47 and Calreticulin-Induced Phagocytosis
Source: ACS Appl Mater Interfaces. 2023 Jan 12;15(3):3791–803. doi: 10.1021/acsami.2c19311 (PMC9880957; doi:10.1021/acsami.2c19311)
Supplement: Supplementary file 1 — am2c19311_si_001.pdf [file am2c19311_si_001.pdf]

Supporting Information

Elimination of cancer cells in co-culture: Role of different nanocarriers in regulation of CD47 and calreticulin induced phagocytosis

Eman M. Hassan[1], Samantha McWhirter[2], Gilbert C. Walker[2], Yadienka Martinez-Rubi[3], and Shan Zou[1,4]*

[1] Metrology Research Centre, National Research Council of Canada, Ottawa, K1A 0R6, Canada;
[2] Department of Chemistry, University of Toronto, 80 St. George St., Toronto, Ontario M5S3H6, Canada
[3] Security and Disruptive Technologies, National Research Council Canada, 100 Sussex Drive, Ottawa ON K1A 0R6 Canada
[4] Department of Chemistry, Carleton University, 1125 Colonel By Drive, Ottawa, ON K1S 5B6, Canada
*Corresponding Author: Shan Zou, Metrology Research Centre, National Research Council Canada, Canada. Phone: 613-949-9675, email: Shan.Zou@nrc-cnrc.gc.ca (ORCID: 0000-0002-2480-6821)

Characterization of graphene oxide, boron nitride nanotube and nanocarriers

Single layered graphene oxide was previously synthesized[S1] using the modified Hummers method. AFM, Raman and XPS characterization, with toxicity assessment were carried out and reported previously.[S2-4] Further details of preparation of GO nanocarrier can be found in the supporting information of ref. S5.[S5] The confirmation of successful loading of siRNA was measured by the changes of surface charges from positive potential values to around -18 to -20 mV by the dynamic light scattering.

Characterization of boron nitride nanotubes and polymer modification were following previously published protocols.[S6] Purified BNNTs were modified with poly(3-methoxy tetraethoxy methyl thiophene) and dispersed in water to make a 0.5 mg/mL BNNT-polymer (BNP-long) dispersion. The dispersion was bath sonicated for 300 mins to obtain BNP-short dispersion. Absorption and AFM characterization were carried out and results are included in Figure S5 and S6. Dispersed BNP (in water) was further diluted in cell media to form stable dispersions (Fig. S5A,B). Fluorescence live cell imaging (Fig. S5C) also illustrated the rapid uptake of BNPs. The successful formation of the BNP-siRNA complex was confirmed by the observed changes of surface charges (changes from positive or neutral potential values to around -17 mV and -18 mV for long-BNP_siRNA and short-BNP_siRNA, respectively).

Quantification of siRNA Measurements

siRNA encapsulated in LNPs was quantified by Quanti-T RiboGreen RNA assay (Life Technologies, Burlington, ON, Canada). Briefly, LNP-siRNA was incubated at 37°C for 10 min in the presence or absence of 1% Triton X-100 followed by the addition of the RiboGreen reagent. The fluorescence intensity (Ex/Em: 480/520 nm) was determined using a plate reader (BMG CLARIOstar Plus). The encapsulation efficiency values for the control siRNA-LNP and CD47_siRNA-LNP were 77% and 75%, respectively.

Other Control Measurements

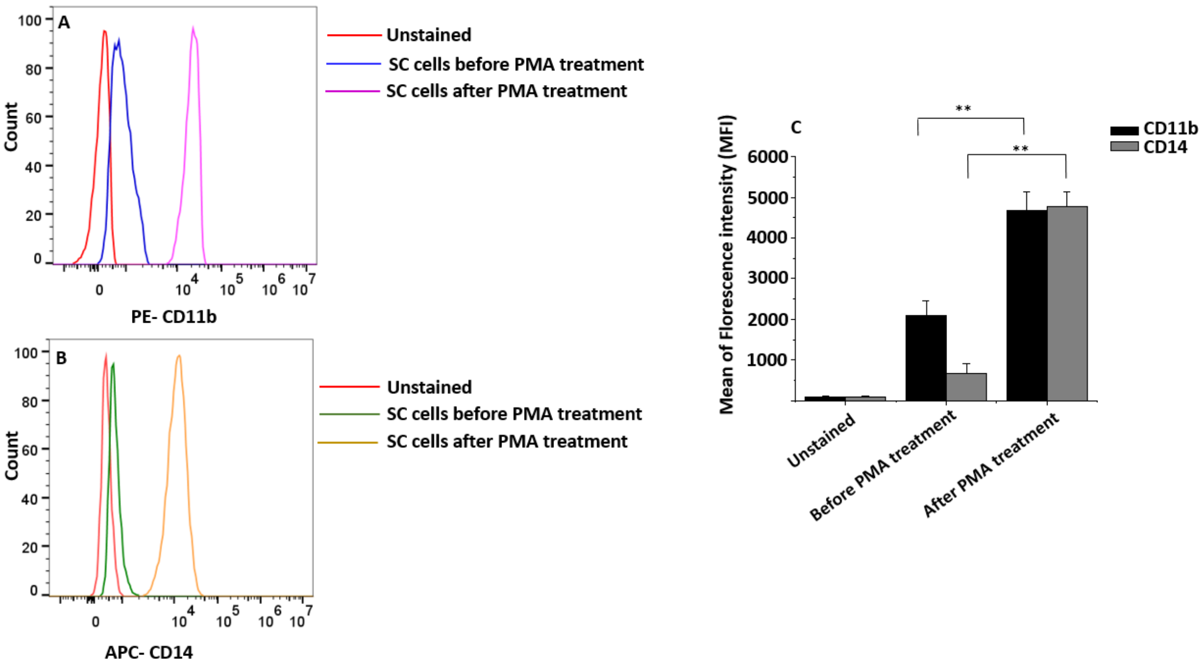

Figure S1. Flow cytometric analysis of human macrophages before and after treatment of PMA. The expression levels of CD11b (A) and CD14 (B) were measured by incubating PE labelled anti-CD11b and APC labelled anti CD14 antibodies with non-treated and PMA treated human macrophages. C: Mean fluorescence intensity (MFI) values of CD11b+ and CD14+ indicate the change in CD11b (A) and CD14 (B), levels before and after PMA treatment. Values in the graphs are shown as means ± SEM of three trials of duplicate samples (n=6). The statistical significance was determined by independent two-sample t test with the note ** $P < 0.01$. SC cells: Human normal monocyte/macrophage.

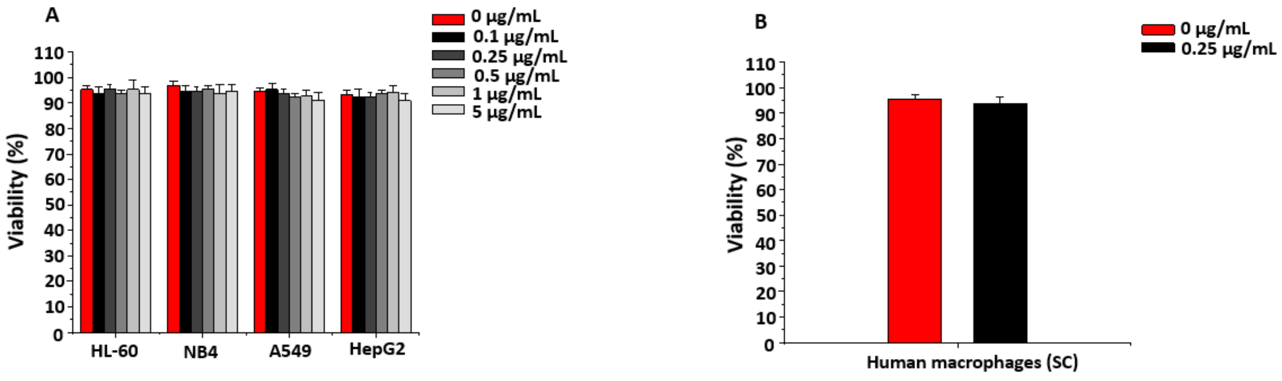

Figure S2. Viability of cancer cells: A: HL-60, NB4, A549 and HepG2 when incubating at different concentrations of small-GO-PEG-PAMAM. B: Viability of human macrophages at 0.25 µg/mL of small-GO-PEG-PAMAM. All cell were incubated with 0, 0.1, 0.25, 0.5, 1, and 5 µg/mL and human macrophages were incubated with 0.25 µg/mL of small-GO-PEG-PAMAM for 48 h in the CO_2 incubator. Then viability of all cells were measured using PE-Annexin V apoptosis detection kit following the manufacturer instructions.

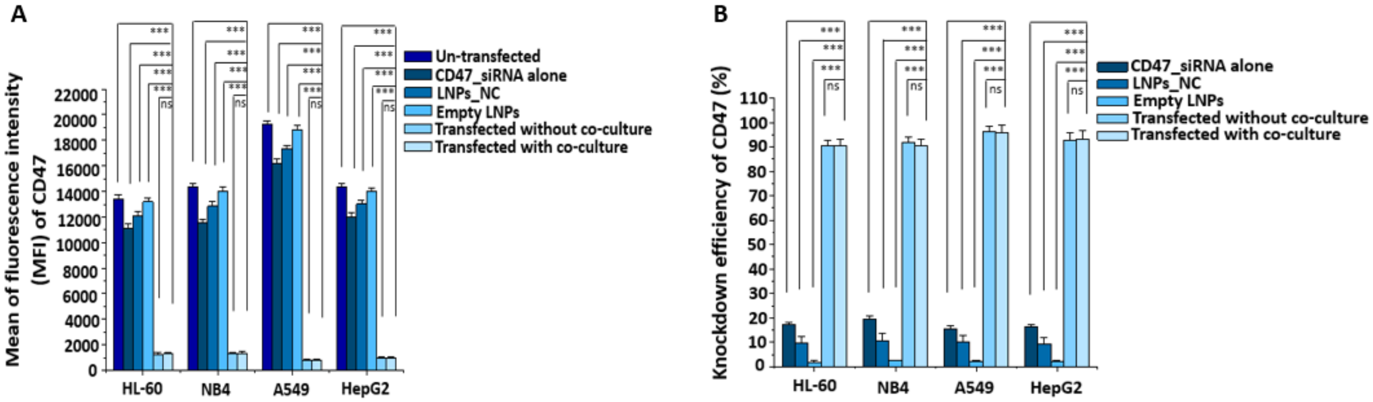

Figure S3. Knockdown efficiency of cancer cells after transfection of CD47_siRNA encapsulated in lipid nanoparticles (LNPs). A: mean of fluorescence intensity (MFI) of CD47 protein levels in all cell lines before and after transfection of CD47_siRNA- LNPs. B: the knockdown efficiency of CD47 protein in all cell lines used after transfection. The statistical analysis of the MFI was determined by one way ANOVA, measured on untransfected, CD47_siRNA alone, LNPs-negative control (NC), empty LNPs, and transfected with and without co-culture in each cell line in (A) and CD47 knockdown efficiency in (B), with the notes of *** P < 0.001 and "ns" not significant.

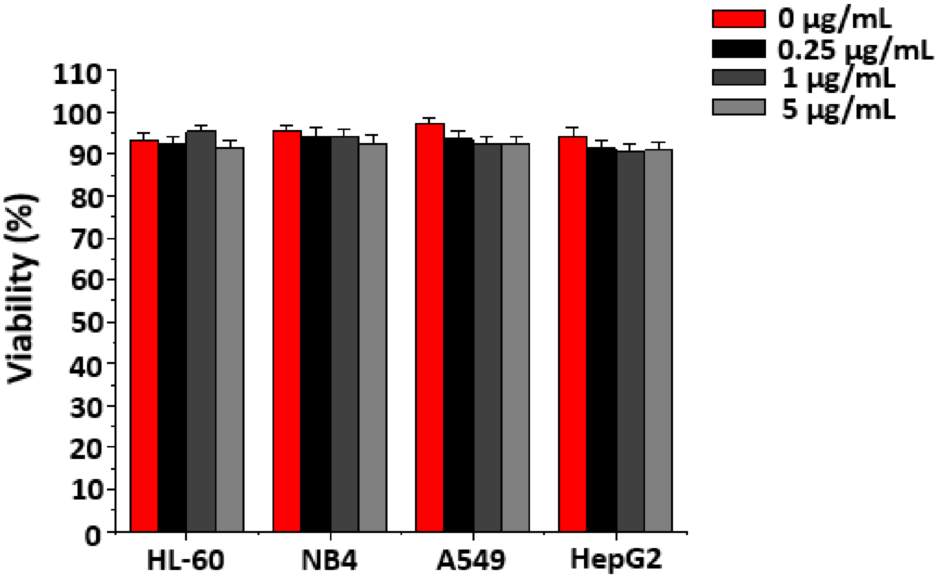

Figure S4. Viability of HL-60, NB4, A549, and HepG2 cell lines when incubating at different concentrations of lipid nanoparticles. All cell lines were incubated with 0, 0.25, 1, and 5 μg/mL of lipid nanoparticles for 48 h in the CO_2 incubator. Then viability of cells were measured using PE-Annexin V apoptosis detection kit following the manufacturer instructions.

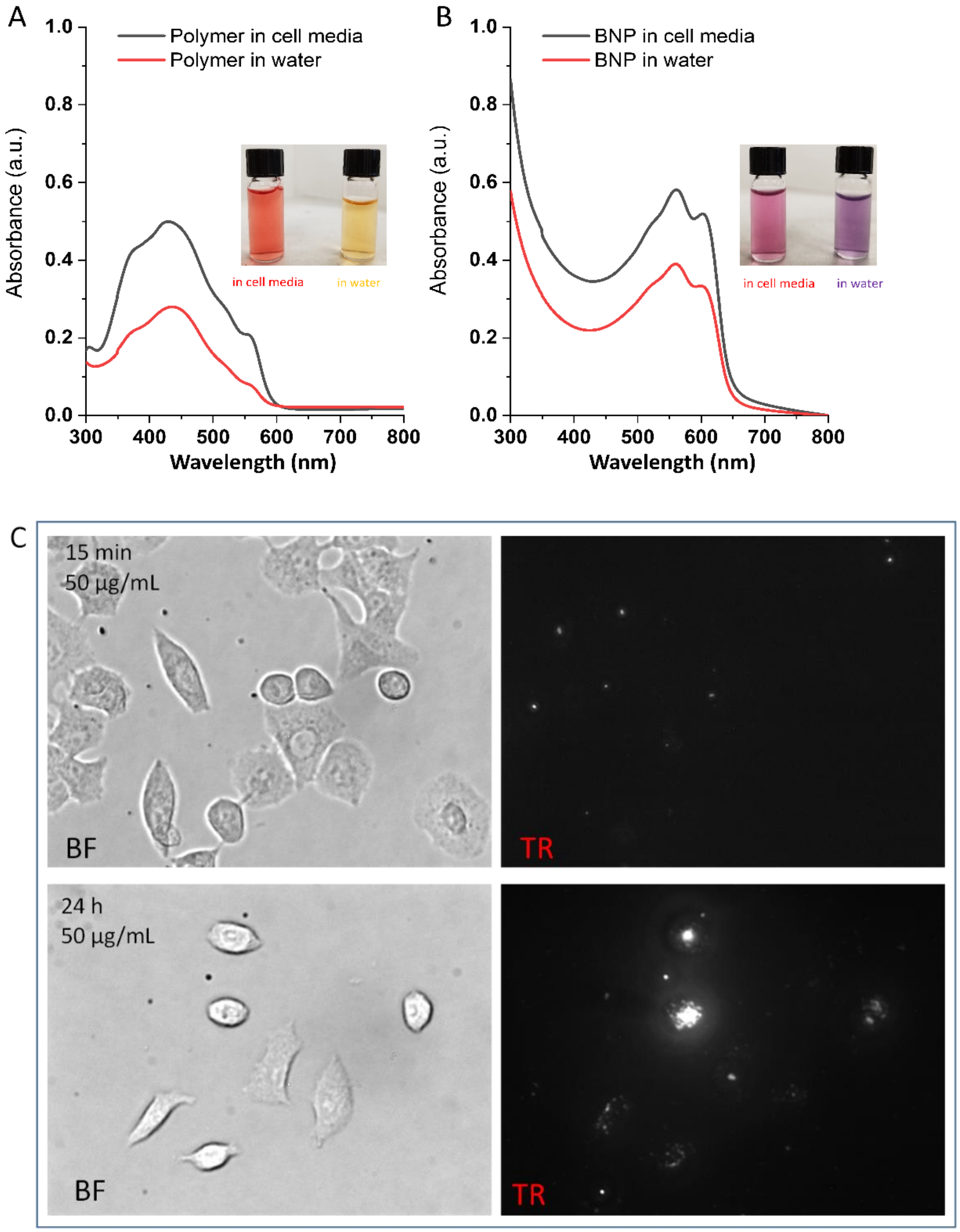

Figure S5. Absorption spectra of poly(3-methoxy tetraethoxy methyl thiophene) (A) and polymer modified boron nitride nanotubes (BNP) (B) in water and cell media. Live cell imaging of BNP treated HepG2 cells (C). BF: Bright field images; TR: Fluorescence images captured using TexasRed filter (image field of view is about 224 µm by 168 µm).

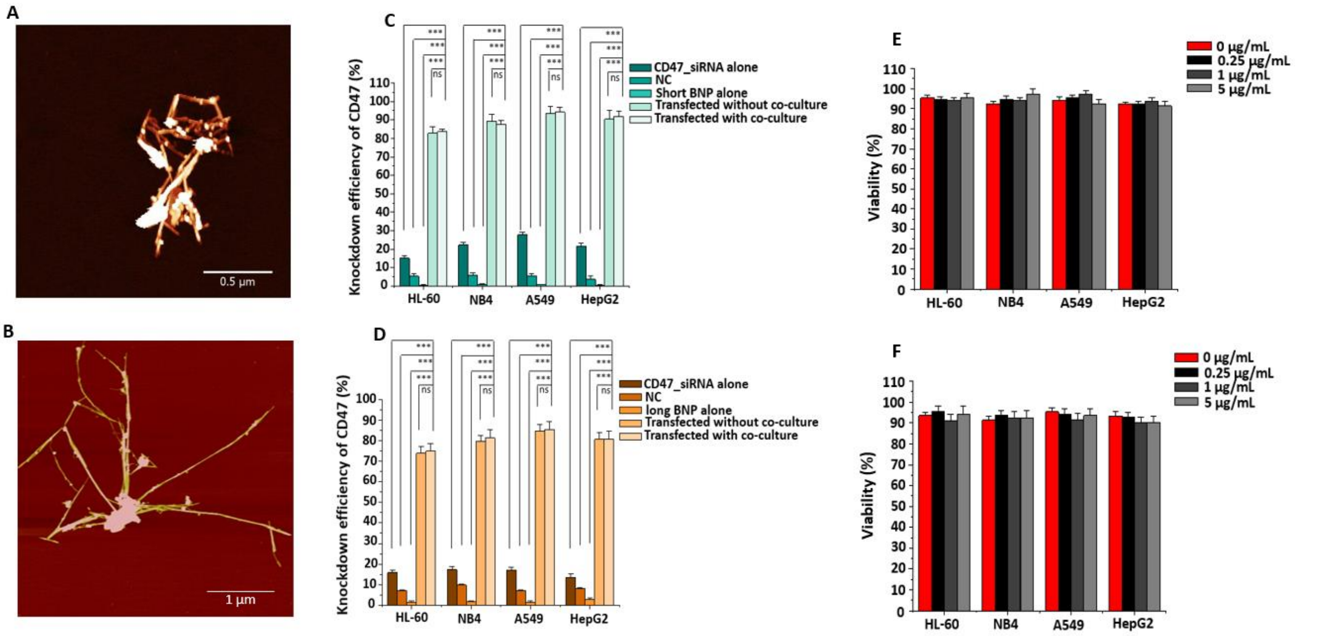

Figure S6. Transfection evaluation of CD47_siRNA conjugated to short and long BNP. A-B: verification of the sizes of short (A) and long (B) BNP using AFM. C-D: knockdown efficiency of CD47 protein after transfection of all cancer cell lines using short (C) and long (D) BNP as nanocarriers. E-F: Viability (%) of all cancer cell lines when incubating at different concentrations of short (E) and long (F) BNP. The statistical analysis of the knockdown efficiencies was determined by one way ANOVA, measured on CD47_siRNA alone, negative control (NC), short and long BNP alone, and transfected with and without co-culture in each cell line in (C) and (D) with the notes of *** $P < 0.001$ and "ns" not significant.

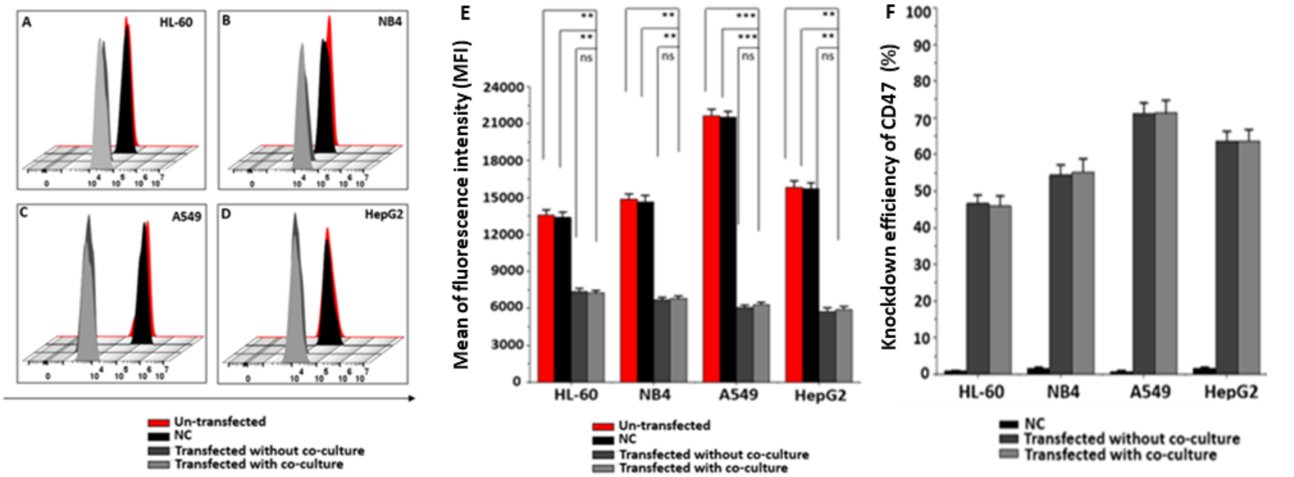

Figure S7. Knockdown efficiency of cancer cells after transfection of CD47_siRNA using lipofectamine RNAiMAX as carrier. (A-D): Overly flow cytometric histograms representing the levels of PE-labelled CD47 protein before and after knockdown of CD47 in HL-60 (A), NB4 (B), A549 (C), and HepG2 (D). E: mean of fluorescence intensity (MFI) of CD47 protein levels in all cell lines before and after transfection of CD47_siRNA- lipofectamine RNAiMAX. F: the knockdown efficiency of CD47 protein in all cell lines used after transfection. The statistical analysis of the MFI was determined by one way ANOVA, measured on untransfected, lipofectamine RNAiMAX -negative control (NC), and transfected with and without co-culture in each cell line in (E) and CD47 knockdown efficiency in (F), with the notes of ** $P < 0.05$, *** $P < 0.001$ and "ns" not significant.

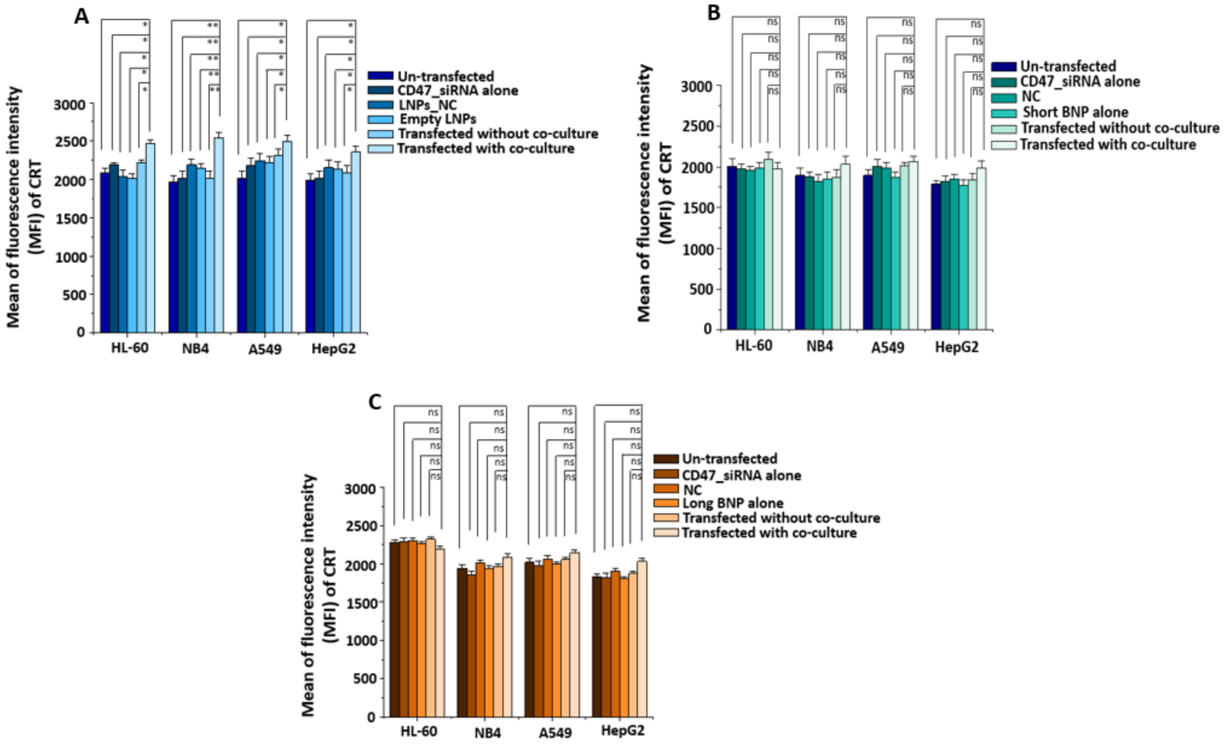

Figure S8. Mean of fluorescence intensity (MFI) of CRT protein in cancer cells. A-C: MFI of CRT protein in HL-60, NB4, A549, and HepG2 cancer cells using LNPs (A), short (B) and long (C) BNP as nanocarriers to deliver CD47_siRNA inside the cell. Cells were harvesting and CRT was measured using Alexa Fluor® 488 antibody. Then cells were analyzed using a flow cytometry. The statistical analysis of the MFI was determined by one way ANOVA, measured on untransfected, CD47_siRNA alone, LNPs-negative control (NC) and NC with short and long BNP, empty LNPs, and short and long BNP alone and transfected with and without co-culture in each cell line in (A-C) , with the notes of * $P<0.05$, ** $P < 0.01$ and "ns" not significant.

References:

S1. Gies, V.; Lopinski, G.; Augustine, J.; Cheung, T.; Kodra, O.; Zou, S., The impact of processing on the cytotoxicity of graphene oxide. *Nanoscale Adv.* **2019**, *1*, 817-826.

S2. Coleman, B. R.; Knight, T.; Gies, V.; Jakubek, Z. J.; Zou, S., Manipulation and quantification of graphene oxide flake size: Photoluminescence and cytotoxicity. *ACS Appl. Mater. Interfaces* **2017**, *9*, 28911-28921.

S3. Gies, V.; Zou, S., Systematic toxicity investigation of graphene oxide: Evaluation of assay selection, cell type, exposure period and flake size. *Toxicol. Res*. **2018**, *7*, 93-101.

S4. ASTM International. E3220-20 Standard Guide for Characterization of Graphene Flakes. West Conshohocken, PA, **2020**. doi: 10.1520/E3220-20.

S5. Hassan, E. M.; Zou, S. Novel nanocarriers for silencing anti-phagocytosis CD47 marker in acute myeloid leukemia cells. *Colloids Surf. B Biointerfaces* **2022,** *217*, 112609.

S6. Martinez Rubi, Y.; Jakubek, Z. J.; Chen, M.; Zou, S.; Simard, B. Quality Assessment of Bulk Boron Nitride Nanotubes for Advancing Research, Commercial, and Industrial Applications. *ACS Appl. Nano Mater.* **2019,** *2*, 2054-2063
